# EDITOR’S MESSAGE / MESSAGE DE LA RÉDACTION

**DOI:** 10.29173/jchla29750

**Published:** 2023-12-01

**Authors:** Megan Kennedy

*[Français en suite]*

Welcome to the December 2023 issue of JCHLA/JABSC! I hope everyone is easing into the colder temperatures and looking forward to a happy and restful holiday season ahead. As the incoming Editor, I would like to thank Colleen Pawlik, former Editor, for her diligent work and her efforts to get JCHLA/JABSC indexed in Web of Science Core Collection as part of the Emerging Sources Citation Index (ESCI). This is a great step forward for the journal and will hugely advance our discoverability and impact (beginning 2024, JCHLA/JABSC will be assigned a Journal Impact Factor!). I also want to thank our outgoing Production Editor, Courtney Ellsworth, for her wonderful work and assistance with onboarding our new Production Editor, Tyler Ostapyk. Finally, I would like to welcome Ashley Farrell who is joining the Editorial Board as Junior Editor.

At the time of writing this message, the Editorial Board is planning to meet for a discussion about "Diversity, equity and inclusion in publishing at Elsevier: a reviewer guide" from Elsevier. This is part of our continued work to educate ourselves on topics related to Equity, Diversity, and Inclusion (EDI). The focus of our discussion will be to review our current peer-review practices and determine where there are areas for improvement in order to be more inclusive to our authors, readers, and reviewers. This work is ongoing so I will continue to share updates as they unfold. I would also like to encourage authors to consider submitting their EDI related work to JCHLA/JABSC.

In this issue, we have a message from the CHLA/ABSC conference planning committee reminding you to get your conference submissions in by January 15, 2024. This issue also includes a program description about the use of Wikipedia in undergraduate health sciences education. Finally, we have two book reviews, *Virtual Services in the Health Sciences Library* and *Knowledge Management: A Practical Guide for Librarians*, and a product review of MeSH on Demand.

Please enjoy the December issue and thank you for your continued support of JCHLA/JABSC!

**Megan Kennedy**

Editor

Email: editor@chla-absc.ca

Bienvenue au numéro de décembre 2023 du JABSC / JCHLA ! J'espère que tout le monde s'accommode des températures plus froides et se réjouit des Fêtes de fin d'année qui s'annoncent heureuses et reposantes. En tant que nouvelle éditrice, j'aimerais remercier Colleen Pawlik, ancienne éditrice, pour son travail assidu et ses efforts pour que JABSC / JCHLA soit indexé dans Web of Science Core Collection dans le cadre de l'Emerging Sources Citation Index (ESCI). Il s'agit d'un grand pas en avant pour la revue, qui améliorera considérablement notre capacité de découverte et notre impact (à partir de 2024, le JABSC / JCHLA se verra attribuer un facteur d'impact de revue !) Je tiens également à remercier notre éditrice de production sortante, Courtney Ellsworth, pour son travail remarquable et son aide à l'intégration de notre nouvel éditeur de production, Tyler Ostapyk. Enfin, j'aimerais souhaiter la bienvenue à Ashley Farrell, qui rejoint le comité de rédaction en tant qu’éditrice débutante.

Au moment de la rédaction de ce message, le comité éditorial prévoit de se réunir pour discuter de "Diversity, equity and inclusion in publishing at Elsevier: a reviewer guide" (diversité, équité et inclusion dans l'édition chez Elsevier : un guide pour les évaluateurs) d'Elsevier. Cette réunion s'inscrit dans le cadre de notre travail continu de formation sur les sujets liés à l'équité, à la diversité et à l'inclusion (EDI). L'objectif de notre discussion sera d'examiner nos pratiques actuelles d'évaluation par les pairs et de déterminer les domaines à améliorer afin d'être plus inclusifs pour nos auteur.trice.s, nos lecteur.trice.s et nos évaluateur.trice.s. Ce travail est en cours et je continuerai à vous tenir au courant de son évolution. J'aimerais également encourager les auteur.trice.s à envisager de soumettre leurs travaux relatifs à l'EDI à JABSC / JCHLA.

Dans ce numéro, nous avons un message du comité de planification du congrès de l'ABSC / CHLA qui vous rappelle de soumettre vos propositions pour le congrès avant le 15 janvier 2024. Ce numéro comprend également une description de programme sur l'utilisation de Wikipédia dans l'enseignement des sciences de la santé au niveau licence. Enfin, nous vous proposons deux critiques de livres, *Virtual Services in the Health Sciences Library* et *Knowledge Management : A Practical Guide for Librarians*, et une critique de MeSH on Demand.

Nous vous souhaitons une bonne lecture du numéro de décembre et vous remercions de votre soutien continu à JABSC / JCHLA !

**Megan Kennedy**

Éditrice

Courriel : editor@chla-absc.ca

